# Generativity Among Japanese Elderly: Its Association With Demographic Characteristics and Health Indicators

**DOI:** 10.1093/geroni/igaa057.1363

**Published:** 2020-12-16

**Authors:** Sachiko Murayama, Erika Kobayashi, Masataka Kuraoka, Kumiko Nonaka, Motoki Tanaka, Yuta Nemoto, Hiroshi Murayama, Yoshinori Fujiwara

**Affiliations:** Tokyo Metropolitan Institute of Gerontology, Tokyo, Japan

## Abstract

Generativity is defined as concern and activity dedicated to contributing to the welfare of others, especially younger generations. Although generativity is postulated to be an important developmental task in old age, there are few reports of its related factors in Asian countries. The purpose of our study is to examine the gender difference of generativity and to explore the defining factors among Japanese elderly. During August to September 2016, we conducted a questionnaire survey for randomly selected 1,187 people aged 65–84 years in the Tokyo area (527 males, 660 females, mean age 72.6 years±5.5), and measured the following variables: generativity, gender, age, length of residence, parental status (having children or grandchildren), working status, commitment to child-rearing activities, mental health (WHO-5 scores), and Instrumental Activities of Daily Living (IADLs). As a result of Student’s t-test, we found that males scored significantly higher on generativity than females (t=2.678, df=1067.097, p<.01). Moreover, we carried out multiple regression analysis, separated by gender. The results showed that, only among males, generativity was positively related to age (β=.096, p<.05) and having children (β=.148, p<.001). In addition, we found that generativity was positively associated with the following variables in both genders: having jobs, commitment to child-rearing activities, mental health, and IADLs (β=.081–.318, p=.000–.032). Among them, mental health and IADLs especially had strong effects on generativity (β=.188–.318, p<.001). We have concluded that the related factors of generativity differ between males and females, but regardless of gender, health indicators are strongly associated with generativity in old age.

